# Pixelated Physical Unclonable Functions through Capillarity-Assisted Particle Assembly

**DOI:** 10.1021/acsami.3c09386

**Published:** 2023-11-01

**Authors:** Zazo Cazimir Meijs, Hee Seong Yun, Pascal Fandre, Geonhyeong Park, Dong Ki Yoon, Lucio Isa

**Affiliations:** † Laboratory for Soft Materials and Interfaces, Department of Materials, ETH Zurich, 8093 Zurich, Switzerland; ‡ Department of Chemistry, Korea Advanced Institute of Science and Technology (KAIST), Daejeon 34141, Republic of Korea

**Keywords:** physical unclonable function, security, capillarity-assisted particle assembly, microparticles, fluorescence, colloidal transfer

## Abstract

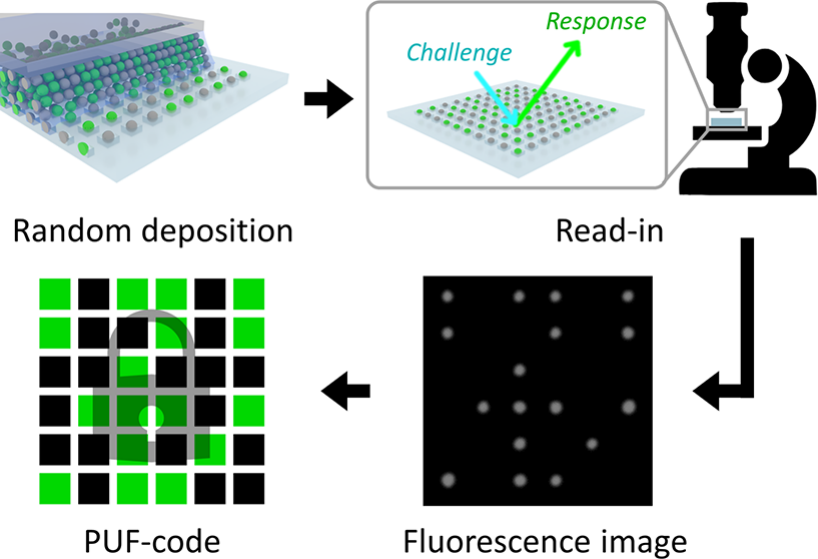

Recent years have shown the need for trustworthy, unclonable, and durable tokens as proof of authenticity for a large variety of products to combat the economic cost of counterfeits. An excellent solution is physical unclonable functions (PUFs), which are intrinsically random objects that cannot be recreated, even if illegitimate manufacturers have access to the same methods. We propose a robust and simple way to make pixelated PUFs through the deposition of a random mixture of fluorescent colloids in a predetermined lattice using capillarity-assisted particle assembly. As the encoding capacity scales exponentially with the number of deposited particles, we can easily achieve encoding capacities above 10^700^ for sub millimeter scale samples, where the pixelated nature of the PUFs allows for easy and trustworthy readout. Our method allows for the PUFs to be transferred to, and embedded in, a range of transparent materials to protect them from environmental challenges, leading to improved stability and robustness and allowing their implementation for a large number of different applications.

## 1 Introduction

Enormous economic damage results worldwide from products that are counterfeited or incorrectly attributed.^1^ Moreover, as in the case of the pharmaceutical industry, in addition to economic damage and violation of intellectual property, counterfeit products can also be highly dangerous.^2^ These challenges have pushed the development of robust strategies to prove the authenticity of products.^3^ An ideal authentication technology must produce truly unique identification tokens that cannot be replicated. Specifically, it is required that even if unauthorized manufacturers or sellers have access to the same techniques, it would still be impossible for them to create identical clones that are associated with a copy of the original product. One excellent way to guarantee these requirements is to use physically unclonable functions (PUFs).^4,5^ A PUF is a physical object that for a given input under certain conditions, also known as the challenge, gives a unique, physically defined output, the response. This response is the unique identifier of the object that is then correctly authenticated. Because the PUF is fabricated by an unpredictable stochastic process containing a random component, it cannot be reproduced by repeating the same fabrication procedure in a viable amount of time.^6^ The characteristics and performance of the PUFs depend on the uniqueness of physical signatures induced by signals such as electronic,^7−9^ optical,^5,10−12^ and radio waves.^13,14^ Among various types of PUFs, optical PUFs, such as randomly scattered fluorescent silk microparticles,^2,10^ speckle patterns,^15^ perovskite systems,^16,17^ liquid crystals,^18,19^ and organic crystals,^20^ have advantages due to their inherent randomness and imperfections in physical materials and manufacturing processes, resulting in high security and entropy.

In general, optical PUFs are fabricated on a micro- or nanoscale to achieve a high information density, identifying a specific region of a large random pattern as a region of interest to be quantified as a unique token.^18,21,22^ Upon reading these PUFs, the read image needs to be matched to the original one, i.e., authenticated. Authentication is often enabled by means of alignment markers, which nonetheless always introduce a certain degree of mismatch between the original patterns and subsequent readouts. Small alignment deviations in fact lead to the input and the output images never being exactly the same and being prone to changes over time and mechanical/chemical damage.^23,24^ These limitations have led to multiple efforts to optimize the robustness and stability of the available PUF systems by encapsulation,^25^ improved system design,^26,27^ material development,^28^ response signal amplification,^29,30^ and most commonly, improved evaluation methods.^4,29^

In this work, instead of using a subset image from a larger pattern, we create a pixelated system of clearly distinguishable single colloids. The token consists of a square array of randomly deposited fluorescent colloids, where each of the particles constitutes a single bit. Our strategy presents some advantages compared to the production of PUFs using fluorescent materials reported in previous studies. First, our pixelated PUFs can be easily obtained from commercially available particles without the requirement to synthesize fluorescent materials specific to PUF fabrication.^2,31,32^ Second, embedding the fluorescent dye inside microparticles, which are subsequently encased in a matrix, leads to PUFs that are more mechanically and chemically stable, in contrast to PUFs where the fluorescent material is directly exposed to the outer environment.^17,33,34^ Moreover, since we can use fluorescent particles of the same size, the fluorescence signals acquired in different channels are analogous, so the code required for image processing to obtain the PUF key is relatively simple and fast. Finally, as the colloids are stable and fully distinguishable, our pixelated PUFs have a very low false negative rate with an encoding capacity that scales exponentially with the size of the full token, as will be quantified later. Furthermore, prearranging the particles into pixels allows us to safely transfer the token to different materials without changing the stored data. The colloidal patterns are created using capillarity-assisted particle assembly (CAPA), a well-established method for colloidal assembly,^35−37^ in which a droplet of a suspension containing a mixture of different types of equally sized colloids is moved over a topographically patterned surface, containing microscopic cavities, or traps, into which the particles are deposited. Patterning the cavities into square arrays directly identifies the areas over which the full tokens are defined, compared to previous continuous patterns, which would require alignment and registration.^38^ As the colloids are uniformly mixed, the deposition of each particle in a trap is a fully random process, and for an equal mixture of *M* types of particles, the number of possible unique tokens scales exponentially with the number of traps (*N*) in the pattern as *M*^*N*^. At a laboratory scale, the deposition of each token takes a few seconds. Even if the process is scaled up to an industrial setting, by choosing an appropriate size of the token, it rapidly becomes statistically unreasonable to reproduce the same pattern within a viable time scale.

We start by introducing the methodology for the fabrication of our pixelated PUFs, followed by a detailed analysis of their randomness and uniqueness. The performance of the PUFs is evaluated by quantifying the bit uniformity, entropy, intra-Hamming distance (Intra-HD) and inter-Hamming distance (Inter-HD), and error rates. The encoding capacity as a function of the key size of the token and the number of particle types is calculated. We moreover show that the encoding capacity does not decrease significantly even if the bit uniformity deviates from an ideal distribution by deriving the probability of reproducing identical PUFs for a nonideal distribution of particle types. The robustness and stability of our pixelated PUFs are challenged by various tests against external stimuli and confirmed via authentication after the tests. We conclude by showing that the PUF tokens can easily be transferred to, and subsequently embedded in, transparent support materials to be potentially used in consumer products.^2,39,40^

## 2 Results

**Figure 1:**
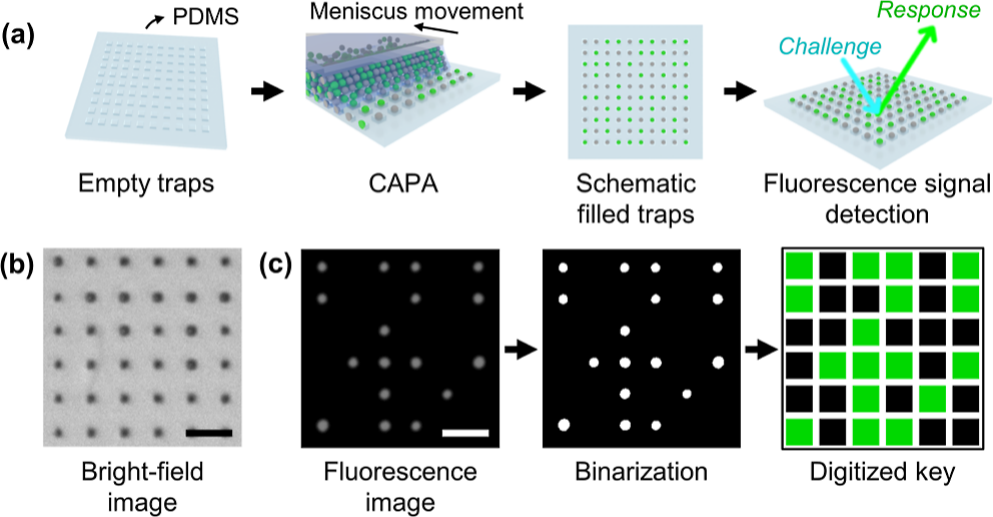
Silica particle array for PUFs. (a) Schematic illustration of the generation of PUFs using CAPA. Nonfluorescent and fluorescent particles are randomly confined by capillary forces in a square array of traps patterned into PDMS. The input challenge, consisting of illuminating the sample with light at the excitation wavelength for the fluorescent particles, results in an output response corresponding to the fluorescence microscopy image of the particle array. (b) Bright-field image of the filled traps after CAPA. (c) Digitization process of the fluorescence microscopy image to obtain the key. A fluorescence microscopy image is binarized and transformed to a digital key by assigning a pixel value corresponding to each trap in the array. All scale bars are 5 μm.

We start by producing *L* × *L* arrays of traps with lateral dimensions 10–20% larger than the diameter of the spherical particles to be deposited and a depth between their radius and diameter, e.g., 1.2 × 1.2 × 0.8 μm^3^ for 1 μm diameter particles (Figure S1), by making a mold through standard two-photon polymerization and replicating it in polydimethylsiloxane (PDMS) (see the Experimental Section).^41^ After the preparation of the trap arrays, we randomly filled the traps with silica particles by means of CAPA (Figure 1a). The particles used in the CAPA are a mixture of nonfluorescent and fluorescent silica particles, all are of 1 μm diameter, have a hydrophilic surface with terminal Si–OH groups giving rise to a strongly negative zeta potential, and are nonporous (see the Experimental Section). In the CAPA process, an evaporating droplet of the particle suspension is dragged at a controlled speed over the template. Evaporation causes the accumulation of the particles at the moving meniscus, which by means of capillary forces pushes the particles inside the traps. Upon depinning of the meniscus, the particles are selectively deposited inside the traps with a controllable yield.^42^ If two or more types of particles of the same size are mixed and no strong interactions between them are present such that they remain colloidally stable, they are randomly distributed into the traps to produce a unique particle array or token. By mixing nonfluorescent particles with green-, blue-, and red-fluorescent ones, we can produce 2-, 3-, and 4-color patterns. The particles used in this work have the same size and a similar negative surface charge (see the Experimental Section for particle details), and we thus do not expect significant segregation effects at the meniscus.^43^ Moreover, it has been previously shown that the capillary forces dominate the deposition process and that electrostatic interactions and corresponding effective particle sizes play a minor role compared to that by the size ratio between the trap and the physical dimensions of the particles.^42^ Because the deposited particles are localized only within the traps, the CAPA automatically leads to the formation of well-defined pixelated patterns.

The simplest PUF produced by this method uses an equal mixture of nonfluorescent and fluorescent, e.g., green-fluorescent, particles. After the CAPA, the two types of particles look the same in the bright-field image (Figure 1b), and the empty traps are indistinguishable from the nonfluorescent particles. Even though the yield of CAPA, i.e., the percentage of filled traps, can be up to 99%, it is beneficial for the robustness of the process that the empty traps do not significantly affect the readout of the PUFs. Upon switching to fluorescence imaging, the location of the fluorescent particles is clearly visualized, and the grayscale fluorescence microscopy image can be easily binarized and converted to the digitized key (Figure 1c). In the final digitized image, the traps containing the nonfluorescent particles or no particles give a value of “0” in the corresponding pixels, while the ones containing the fluorescent particles give a “1”. To read the PUF, the input challenge is thus simply illumination with the corresponding light at the excitation wavelength for the fluorescent particles, and the output response is the fluorescence microscopy image generated by their specific emissions. For each PUF, the response can be safely stored as a digital key, e.g., in a repository of the manufacturer, and any physical token can be tested against this repository for authenticity.

**Figure 2:**
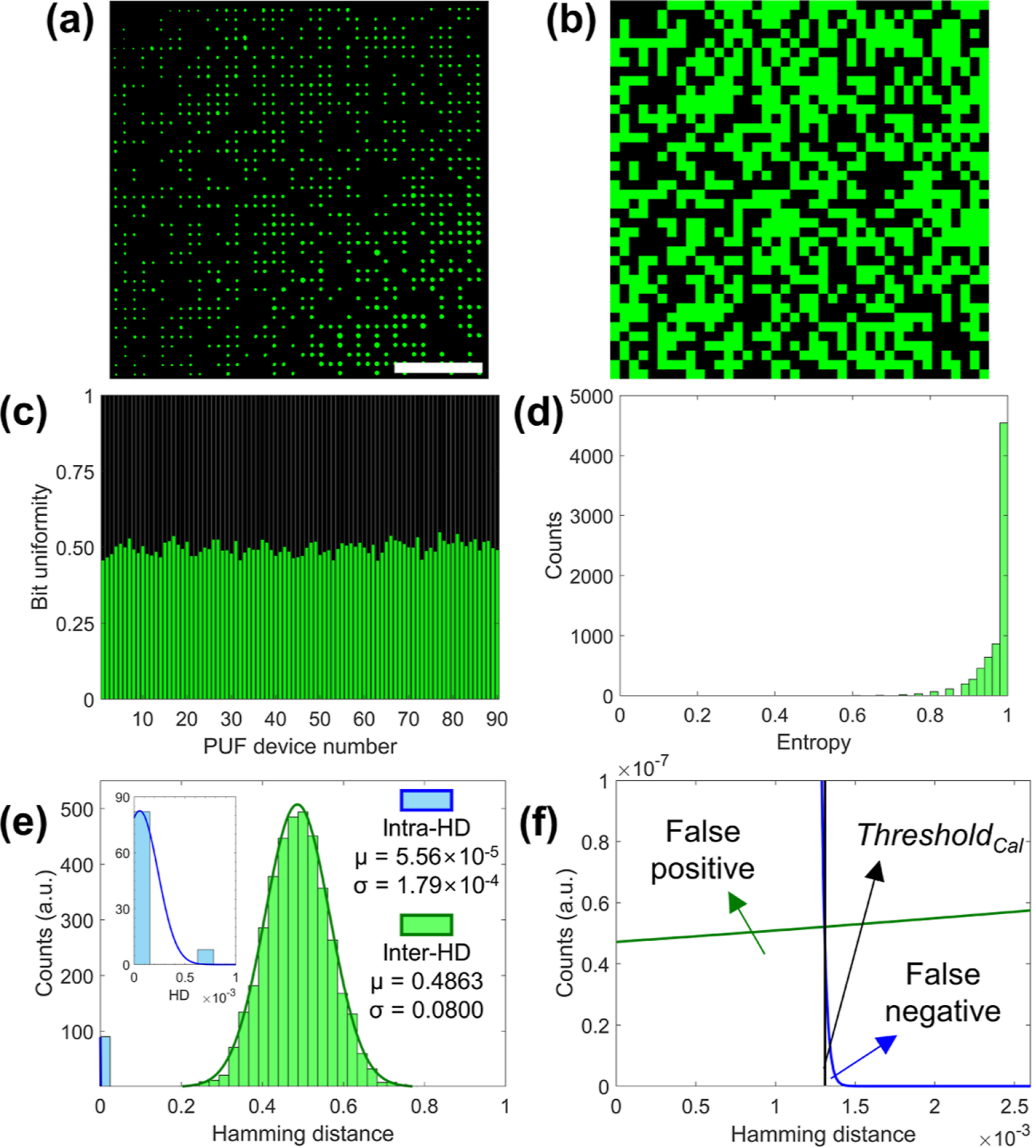
Statistical analanalysis of PUFs. (a) Fluorescence microscopy image (b) and resulting digitized key of a representative PUF fabricated by CAPA using a 50:50 mixture of nonfluorescent and green-fluorescent silica particles. The dimensions of the array are 40 × 40. (c) Distribution of bit uniformity for each PUF over 90 PUFs. (d) Entropy histogram for the same 90 PUFs. (e) Histogram of the Intra-HD and Inter-HD fitted with Gaussian distributions. Inset: zoomed-in view to visualize the Gaussian fitting of Intra-HD. (f) Schematic diagram of how to calculate the false positive rate, false negative rate, and threshold of authentication. Scale bar in (a) is 50 μm.

We evaluated 90 different 40 × 40 2-color PUF tokens generated by CAPA, of which an example is given in Figure 2a,b before and after digitization, respectively. As each trap is randomly filled with one of the particles in the mixture, for a 50:50 ratio, the probability that each trap is filled with one particle type is simply 50%. As the deposition of each particle in a trap is an independent event, we expect each pattern to be a random sequence with an average of 50% of the traps being filled by each particle type, as demonstrated in Figure 2c.

The randomness of the PUF tokens is measured by several statistical tests. Most simply, the distribution 1- or 0-bits in the array can be measured to evaluate bit uniformity
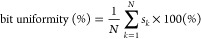
1where *s*_*k*_ is *k*-th bit, which is 0 or 1, for a 2-color PUF-key and *N* = 1600 for a 40 × 40 PUF token (*L* = 40). Figure 2c shows that the average bit uniformity is 51.96 ± 0.19%. The distribution of bit uniformity follows a binomial distribution, which can be described by a Gaussian distribution for a large number of colloids, following the central limit theorem. The randomness of our PUFs is further characterized in Figure 2d through the distribution of the Shannon entropy (*E*) values for each line or column of each token, defined as^44,45^
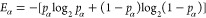
2where α indicates either the *x*- or *y*-axis of the array and *p*_α_ is the probability of finding a value of “1” along the *x*- or *y-*axes. For an ideal uniform mixture of the two particles, all  and . In the case of our experiments, the distribution of the entropy values strongly peaked at 1, showing that our PUFs have clear randomness. The average entropy values along the *x*- and *y*-axes of one PUF in Figure 2b are 0.9672 ± 0.0477 and 0.9756 ± 0.0340, respectively (Figure S2). For the overall ensemble of sequences corresponding to the *x*- and *y*-axes of 90 PUFs, the average entropy value is 0.9714 ± 0.0416. Finally, we evaluate the uniqueness of our PUFs by examining the histogram of the normalized Hamming distance (HD) (Figure 2e). To evaluate whether the same PUF key can be obtained when the same sample is remeasured, the Intra-HD is calculated (inset of Figure 2e). To get the uniqueness, indicating how different PUFs can be distinguished, the Inter-HD is calculated. If *S*_*i*_ = (*s*_*i*,1_, *s*_*i*,2_, ..., *s*_*i*,*N*_) is defined as the *L* × *L* = *N*-bits sequence of the *i*-th PUF, HD is mathematically given by^46,47^
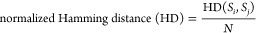
3where
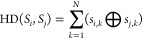
4

If *s*_*i*,*k*_ and *s*_*j*,*k*_ are the same, *s*_*i*,*k*_ ⊕ *s*_*j*,*k*_ = 0. If not, *s*_*i*,*k*_ ⊕ *s*_*j*,*k*_ = 1. The indices *i* and *j* indicate the *i*-th and *j*-th PUF key, respectively, *q* is the number of evaluated PUF keys, and *N* is the number of bits in each PUF key, i.e., for Figure 2, *q* = 90 and *N* = 1600. In particular, two sequences produced by measuring the same PUF token (*i* = *j*) twice are used to determine the Intra-HDs. In contrast, the Inter-HDs are calculated using two binary sequences (*i* ≠ *j*) that were collected from many PUF tokens. Since the histograms in Figure 2e were obtained using 90 PUF tokens, each histogram for the Intra-HD and Inter-HD contains 90 and _90_*C*_2_ (=(90 × 89)/2 = 4005) data points, respectively.
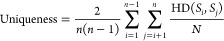
5

From the reported data, the uniqueness of our PUF is 0.4976, corresponding to the mean value of the Inter-HD distribution plotted in Figure 2e, which is very close to the ideal uniqueness for a system of independent events with two equally likely options, which is 0.5, indicating that each PUF is highly distinguishable from the other PUFs. In order to calculate the threshold value required for the authentication process, the histograms of the Intra-HDs and Inter-HDs are each fitted to a Gaussian distribution (Figure 2e), and the threshold for true authentication is determined as the overlapping point of the two Gaussian distributions. The calculated threshold value (Threshold_Cal_) of the HD is 1.3 × 10^–3^ (Figure 2f).

For practical use, the same PUF must be proven to be genuine even after multiple measurements, and different PUFs must be distinguishable. To quantify the occurrence of these events in our case, we examined two error rates. The false positive rate (FPR) is the probability of recognizing a fake PUF as authentic (Type-I error), and the false negative rate (FNR) is the probability of identifying an authentic PUF as fake (Type-II error). The FPR and FNR determined by Threshold_Cal_ are calculated based on the Gaussian distributions of the Inter-HD and Intra-HD reported in Figure 2f. In our case, the FPR is 6.705 × 10^–10^ and FNR is 1.307 × 10^–12^, respectively, showing that the probability of erroneous recognition is very small.

Because image quality may vary under different conditions in real situations, we also carry out a test of the authentication process in the presence of an artificial digital noise, as may be found in situations of low illumination levels or for cameras with low sensitivity. We artificially add pixel-to-pixel noise to a fluorescence microscopy image and verify up to which point the presence of a particle in a trap, giving a “0” or a “1” in a given pixel, can be verified. We set the threshold for authentication to have an FPR of 5 × 10^–9^ and an FNR of 0, corresponding to a threshold value of the HD of 0.0278. Up to the noise level of 0.05, as described in the Supporting Information, the true positive rate (TPR) is 100%, but for noise levels greater than 0.10, the TPR rapidly decreases (see Figure S5 for details). The TPR at higher noise levels can be increased by adding an intermediate step in image processing to filter out the digital noise or enhance the detection of the particles.

The time required for enrollment and authentication is determined by imaging and image processing time. We obtained the enrollment time for the 90 PUF tokens in Figure 2 as the time it takes for the raw fluorescence microscopy images to be converted into 40 × 40-pixel digital keys and stored. The average and standard deviation of the enrollment time for one PUF token is 0.87 ± 0.03 s. The authentication time is measured using 10,000 artificial PUF keys in the database. The time taken to compare one PUF key to be authenticated with another PUF key in the database is 0.524 ± 0.078 ms using a MATLAB serial operation. Using serial operations, the overall authentication time increases geometrically with the number of keys in the database. However, the authentication time can be linearly decreased in proportion to the number of computing units by parallelizing the authentication operation.

**Figure 3:**
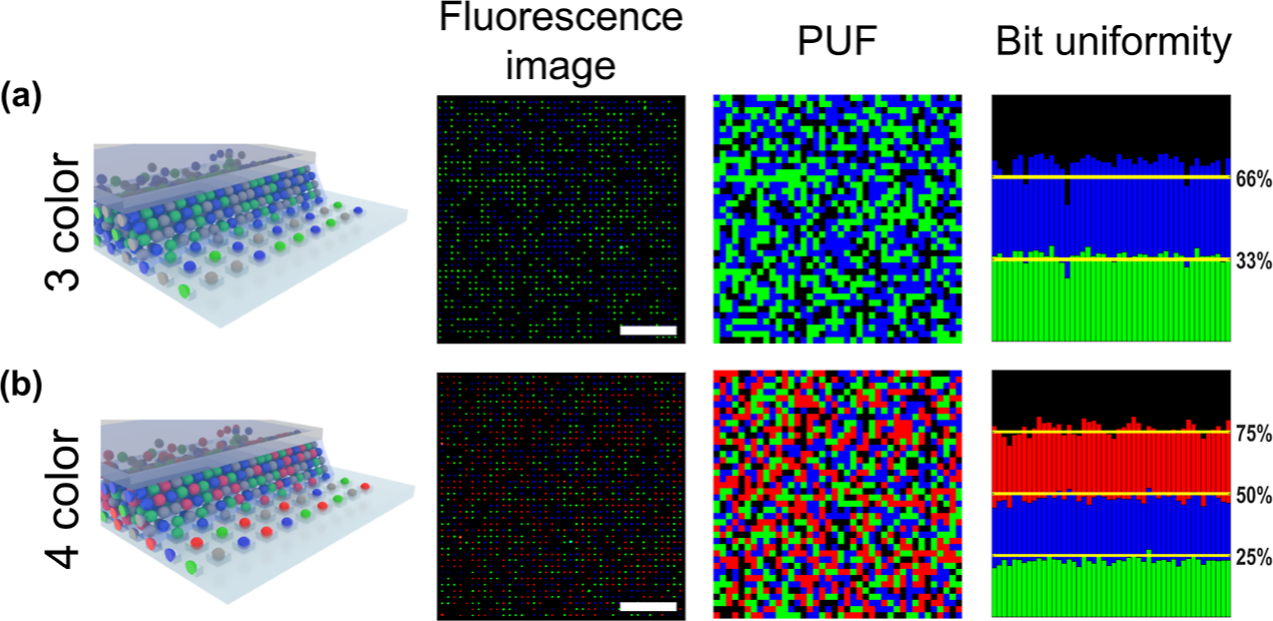
Multicolor PUFs. The first column shows a schematic of the CAPA process. The second one displays a representative fluorescence microscopy image, obtained by overlaying all of the relevant fluorescence channels. The third one reports the corresponding digitized PUF. The fourth column shows the bit uniformity distribution for 50 independent PUF keys. (a) 3-color PUF from a 33:33:33 suspension of green, blue, and nonfluorescent particles. (b) 4-color PUF from a 25:25:25:25 suspension of green, blue, red, and nonfluorescent particles. Array dimensions are 40 × 40. All scale bars are 50 μm.

The same concept introduced above can be extended to *M* different types of particles, where, for equal mixtures, the probability to fill one trap with a given “color” becomes 1/*M*. Examples of PUF tokens with 3 and 4 colors are reported in Figure 3a,b, respectively. In those cases, the digitization of the image follows from the binarization of each separate fluorescence channel and pixelated “RGB” tokens are readily obtained. We observe that a high bit uniformity is retained in the 3- and 4-color systems, following the randomness of the deposition process.

**Figure 4:**
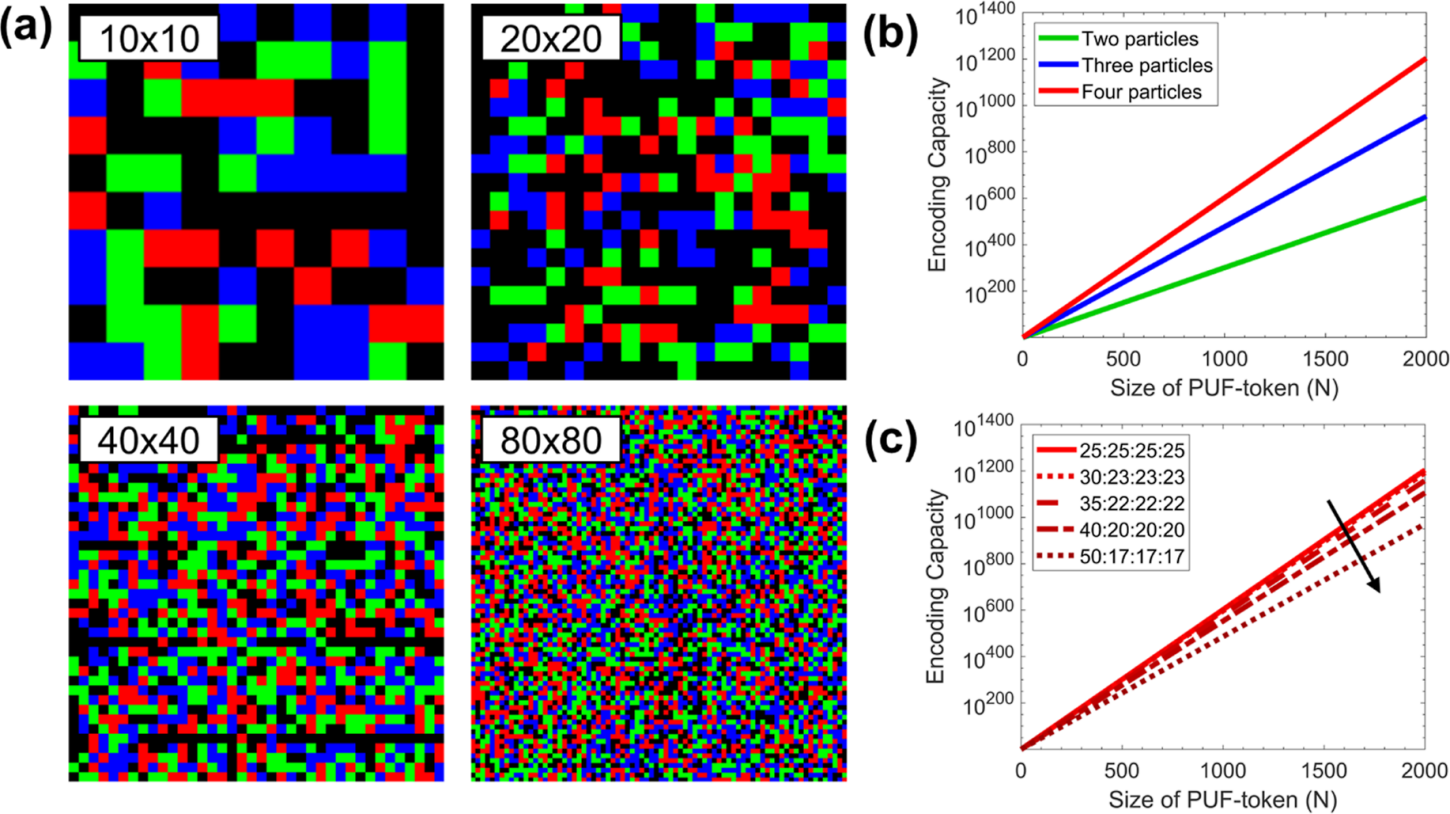
Effects of token size. (a) Examples of digitized 4-color PUF-tokens for 10 × 10, 20 × 20, 40 × 40, and 80 × 80 traps. (b) Plot of the encoding capacity for equiprobable depositions of mixtures of two, three, and four particle types and (c) for several mixing ratios for 4-color as a function of the size of PUF token (*N*) following eq 7.

The generation of PUF tokens by means of CAPA affords an easy route to increase the encoding capacity of each token simply by increasing the size *L* of the particle array. The encoding capacity is generally defined as the number of distinct patterns that can be generated with the PUF method.^4,6,47^ For a completely random deposition of an equal mixture of *M* particle types, the filling of each trap with a given particle type is equiprobable, and the probability of reproduction is simply one over the total encoding capacity *M*^*N*^. Examples of 4-color patterns with different array sizes are given in Figure 4a for 10 × 10, 20 × 20, 40 × 40, and 80 × 80 arrays, corresponding to the physical sizes of 48 × 48 μm^2^, 100 × 100 μm^2^, 204 × 204 μm^2^, and 412 × 412 μm^2^, respectively. The tokens can be evaluated in a single field of view of the microscope. Taking the case of a 40 × 40 array, the system reaches an encoding capacity of 4^1600^ ≈ 10^963^. Considering that depositing particles over a single array line takes approximately 1 s, it is apparent that the time scales to repeat a given random pattern become unrealistic for arrays containing more than a few lines. The encoding capacity for PUF tokens with different numbers of kinds of particles is plotted in Figure 4b. Another significant advantage of using prepixelated PUF tokens is the robustness of the process in terms of the encoding capacity relative to different particle mixing ratios. As we prepare our particle suspensions with an equal mixture of the different types of particles, we can expect the probability associated with their deposition to be equal. However, even in the optimal deposition conditions,^48^ an overall deposition yield below 100% is found, leading to the fact that “0” pixels, corresponding to both empty traps and nonfluorescent particles, may be over-represented.

While the total number of possible patterns is still the same, and thus the total encoding capacity is conserved, the different patterns are no longer equiprobable. In this case, one should define the effective encoding capacity, which corresponds to the inverse of the probability of recreating the same state. In the case of two types of particles, we can describe the probability of repeating the same filling of a single trap as the sum of the probabilities that it is either empty and nonfluorescent particles twice, i.e., *p*^2^ or green twice, i.e., (1 – *p*)^2^. From this, it follows that the probability of repeating the sequence of *N* events is simply the multiplication of each deposition being the same twice, and thus
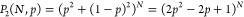
6

This estimation can be extended to the case of 3 and 4 different particle types. So, we can calculate the approximate probability of reproduction in the 4-color case as
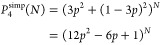
7

In Figure 4c, we plot the effective encoding capacities for different distributions. We see that even for a 40:20:20:20 distribution for a 40 × 40 token (*p* = 0.2, *N* = 1600), the encoding capacity is ≈10^763^ letting us confidently state that the system is robust against deviations from ideal bit uniformity for reasonably sized tokens. Further details of the derivation can be found in the Supporting Information.

**Figure 5:**
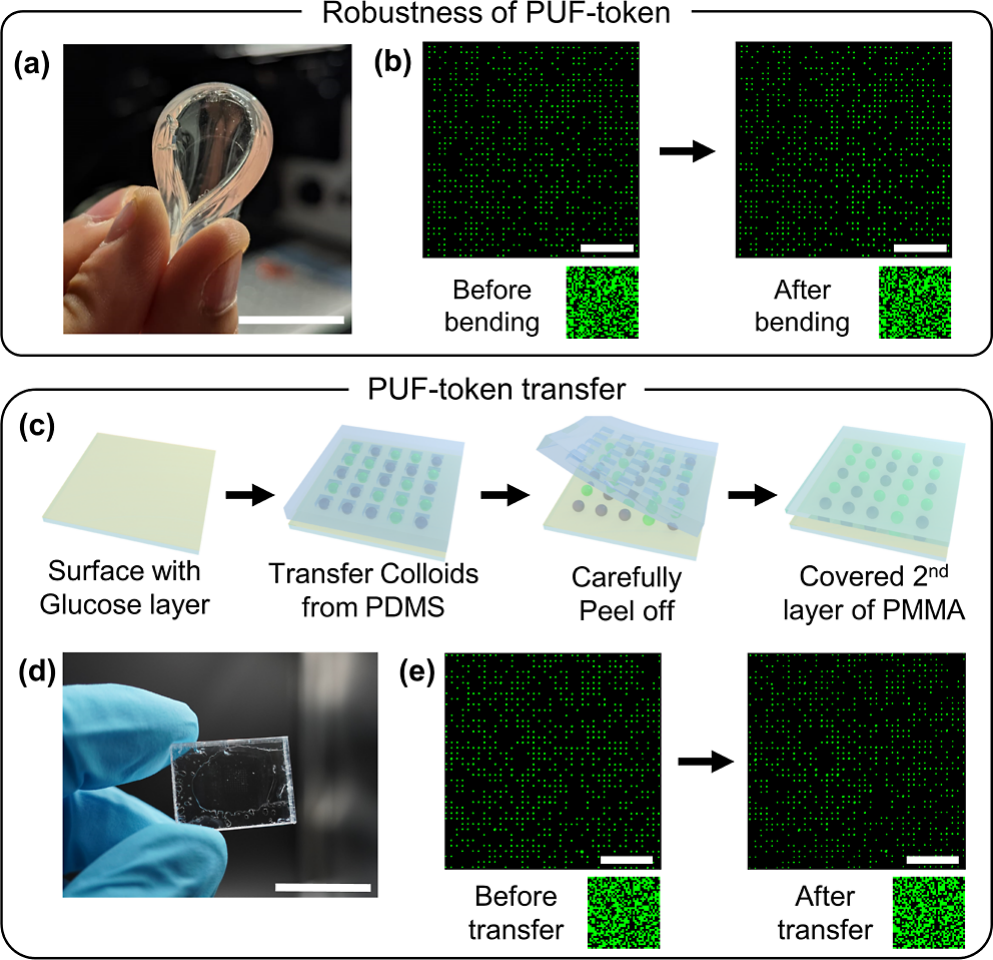
Produced PUF tokens can be encased allowing for protection against many environmental factors. (a) Example of the resistance to bending of a PUF token fully encased in PDMS. (b) Fluorescence microscopy images and corresponding key images of an encased PUF token before and after bending test (more results for various tests are provided in Figure S6). (c) Scheme of the token-transfer process. (d) Photograph of a PUF token transferred to PMMA. (e) Fluorescence microscopy image of a 40 × 40 2-color PUF token before and after transfer to a PMMA layer. Scale bars in (a), (d) are 2 cm, and (b), (e) are 50 μm.

Finally, the inherent pixelated nature of our PUF tokens allows for pattern recognition without requiring alignment marks and enables measurement with any standard fluorescence microscope. Moreover, after deposition, the particle patterns can be fully embedded into transparent elastomers or transferred to different transparent supports. Encapsulations enable the protection of the token against environmental damage, and the resistance of the token against different damaging agents then depends on the material into which it is transferred. For example, a second layer of PDMS can be cast over the CAPA template to fully embed the PUF token within a protective elastomeric casing. PDMS is transparent to visible light,^49^ allowing encapsulation without limiting the accessibility to the fluorescence signals. Encapsulation in PDMS protects the PUF token against disruption by many actions, as tested by 100 bending cycles (Figure 5a,b), impact and pressure up to 4 N/μm^2^, and stretching of up to 20% (Figure S6). Moreover, the pattern remains intact after exposure to external stimuli like heating to 200 °C, immersion in ethanol and acetone for 30 min, exposure to UV light for 1 h, and exposure to daylight for up to 3 days (Figures S6–S8 for more details). The robustness to various stimuli and the photostability of our PUFs are quantitatively confirmed through Intra-HD before and after the test and the corresponding authentication process. While PDMS is flexible and resistant to a range of solvents, it is severely swollen by many other organic solvents, and it is easily damaged by sharp objects. In order to adapt the stability of the PUF to different conditions, it can also be easily transferred to different substrates, i.e., more rigid ones. As an example, we transferred a token onto a poly(methyl methacrylate) (PMMA) substrate and covered it with another PMMA layer (Figure 5d). The transfer process makes use of an adhesive transfer layer, in this case glucose, and it is adaptable to many different substrates (Figure 5c). This process allows for transferring up to 100% of the initial CAPA-PUF, as shown in Figure 5e, but it is important to note that even if a small fraction of the particles are not transferred, the effective encoding capacity is still sufficient, as presented in Figure 4c.

## 3 Conclusions

We present the use of an established method for particle deposition, CAPA, to produce PUFs. The main advantage of the method lies in the direct fabrication of pixelated QR code-like tokens that can be easily read, stored, and transferred to different materials for applications. The simplicity of our method allows us to easily change the size of the tokens and the number of color channels to suit different applications, i.e., which require small tokens or larger ones for improved security. The method is robust to deposition errors and nonideal distributions of the different particles. The size of the particles makes it extremely hard to deterministically reproduce any specific PUF token, and with the exponential increase in possible tokens, recreating the same token using a random process rapidly becomes statistically impossible. As an example, if it takes 10 s to make and measure a 40 × 40 token with 3 different colloids, it would on average take 0.5 × 3^1600^ × 10 s ≈ 10^360^ s or about 10^350^ years of constantly making tokens to remake the same one. This high encoding capacity for security is kept for imperfect depositions as empty traps are indistinguishable from nonfluorescent particles, and small deviations from an ideal distribution have a minimal effect on the uniqueness of the token. As downscaling of CAPA to nanoscale particles has already been shown,^36^ in the future, we envisage the systematic production of nanometric PUFs, e.g., employing quantum dots or plasmonic nanoparticles for readout. Lastly, the ability to embed the token in other transparent media allows them to be used in a broad range of applications without damage or degradation.

## 4 Experimental Section

### 4.1 Silica Microparticles

All the nonfluorescent and fluorescent spherical silica microparticles were purchased from micromod Partikeltechnologie GmbH. According to the technical notes of the particle supplier, ortho-silicates and related compounds are hydrolyzed to produce nonfluorescent and fluorescent silica particles. All particles have 1 μm average diameter. They have a hydrophilic surface with terminal Si–OH groups and are nonporous. The product details for all particle batches are as follows: nonfluorescent particles - product code: 43–00–103, zeta potential = −63.6 ± 0.3 mV; red-fluorescent particles - product code: 40-00-103, zeta potential = −59.0 ± 0.6 mV, excitation wavelength (λ_ex_) = 569 nm, and emission wavelength (λ_em_) = 585 nm; green-fluorescent particles - product code: 42-00-103, zeta potential = −66.7 ± 0.6 mV, λ_ex_ = 485 nm, and λ_em_ = 510 nm; blue-fluorescent particles - product code: 41-00-103, zeta potential = −63.2 ± 0.3 mV, λ_ex_ = 354 nm, and λ_em_ = 450 nm. The stock number of particles per unit volume for all particle dispersion is 4.8 × 10^10^/mL. All zeta potentials were measured with a ZetaSizer Nano DLS in Milli-Q water at 25 °C. The presented values are averages of 10 measurements, and the error corresponds to their standard deviation.

### 4.2 PDMS Stamp Production

The PDMS templates were produced as imprints of 3D master molds fabricated by two-photon polymerization using a Nanoscribe Photonic Professional GT2 (Nanoscribe GmbH). The molds consist of square areas of *L* × *L* hosting traps of 1.2 × 1.2 × 0.8 μm^3^, with 4 μm spacing between the traps and 200 μm spacing between different arrays, printed on 2.5 × 2.5 cm^2^ fused silica substrates with the standard photoresist IP-Dip2 and a 63× NA 1.4 objective (all printing parameters set to default). The printed masters are developed in propylene glycol monomethyl ether acetate (PGMEA; 99.5%, Sigma-Aldrich) for 10 min and subsequently rinsed with isopropanol. To enhance the adhesion between the print and substrate, the masters were further placed in a UV box for 8 h under 365 nm wavelength light illumination. The masters were silanized via chemical vapor deposition (CVD) with trichloro(1*H*,1*H*,2*H*,2*H*-perfluorooctyl)silane (FOTS; 97%, Sigma-Aldrich) for 20 min under a standard vacuum before pouring PDMS onto them.

The PDMS templates were fabricated using a 10:1 ratio of silicon elastomer precursor and curing agent (Sylgard 184 silicone elastomer kit, Dow Chemical). Mixtures were poured onto the masters and degassed for 10 min. The PDMS was then polymerized overnight in an oven at 75 °C. For PDMS encapsulation, the same procedure was repeated for a sample with particles deposited, giving an approximately 4 μm thick sample with the PUF token approximately in the center.

### 4.3 CAPA

The principles of CAPA have been extensively described elsewhere.^35−37^ In brief, we dispersed the silica particles into a 0.5 mM sodium dodecyl sulfate (SDS) aqueous solution with 0.005 wt % Triton X-45 and a 0.01 wt % suspension of the colloids in the desired ratios. The colloidal particle mixture was vortexed and sonicated for 2 min before use. Around 40 μL of suspension was dragged over the PDMS template at a constant speed of 2 μm s^–1^ by a flat PDMS piece connected to a linear drive motor (Thorlabs DRV014). The deposition was carried out at room temperature (23 °C).

### 4.4 Measurement and Evaluation of PUFs by Fluorescence Microscopy

The PUF tokens were imaged by a bright-field and fluorescence optical microscope. For optical excitation of the red-, green-, and blue-fluorescent particles, green, blue, and ultraviolet light-emitting diodes (LEDs) with wavelengths of 508, 470, and 395 nm were used as light sources, respectively. Band-pass filters of 698 (70), 515 (30), and 432 (36) nm were placed between the light source and the PUF token. The power of the LED was kept constant between each measurement, with an exposure time of 5 s.

Two different microscopes were used in the project. Data for Figures 2 and 3 were recorded with an Imager1 microscope (Zeiss) using a Retiga-6 CCD camera (Teledyne). Data for Figures 4 and 5 were recorded with an Eclipse Ti-2 (Nikon) using an ORCA-Flash4.0 V3 Digital CMOS camera (C13440-20CU, Hamamatsu). All tokens were evaluated using a 40× objective, except the 80 × 80 tokens presented in Figure 3, which were measured with a 20× objective. Each color is an independent black-and-white image with the full token in the frame in the same location. The evaluation and statistical tests were all implemented in MATLAB (R-2020b), and details on the specific code used are provided in Supporting Information Figure S4.

### 4.5 Transfer of PUF Token to PMMA Substrate

A flat PMMA substrate was cleaned by rinsing with ethanol and then exposed to an air plasma. Subsequently, the PMMA substrate was covered with a thin layer of glucose (D-(+)-Glucose; 99.5%, Sigma-Aldrich) by spin-coating, using a 30 wt % water suspension at 4000 rpm for 30 s. The PDMS template with particles trapped by CAPA was carefully stamped on the glucose layer and gently peeled off, leaving the particles on the PMMA substrate. A second PMMA substrate was fixed on top of the sample using a 40 wt % solution of PMMA (*M*_w_ = 15,000, Sigma-Aldrich) in PGMEA (99.5%, Sigma-Aldrich). Finally, it was dried on a hot plate at 80 °C for 10 min to fully fix the PUF token.

## Data Availability

All data needed to evaluate the conclusions in the paper are present in the paper and/or the Supporting Information. Additional data related to this paper may be requested from the authors upon reasonable request.

## Supporting Information Available

SEM images of the PDMS molds and produced tokens after CAPA, additional statistical tests of the two-colloid system corning bit uniformity and entropy, details of the derivation of the effective encoding capacity including discussion on a general distribution, digitization process of the fluorescence microscopy images, test of the authentication under artificial imaging noise, further fluorescence microscopy images for stability tests on the PDMS-encased tokens, and stability of the fluorescent signal under UV light and prolonged daylight exposure (PDF)
