# Supplementary material for: Pixelated Physical Unclonable Functions through Capillarity-Assisted Particle Assembly
Source: ACS Appl Mater Interfaces. 2023 Nov 1;15(45):53053–61. doi: 10.1021/acsami.3c09386 (PMC10658447; doi:10.1021/acsami.3c09386)
Supplement: Supplementary file 1 — am3c09386_si_001.pdf [file am3c09386_si_001.pdf]

Supporting Information:

Pixelated Physical Unclonable Functions through Capillarity-Assisted Particle Assembly

Zazo Cazimir Meijs,[†,¶] Hee Seong Yun,[‡,¶] Pascal Fandre,[†] Geonhyeong Park,[‡] Dong Ki Yoon,[*,‡] and Lucio Isa[*,†]

†*Laboratory for Soft Materials and Interfaces, Department of Materials, ETH Zurich, 8093 Zurich, Switzerland*

‡*Department of Chemistry, Korea Advanced Institute of Science and Technology (KAIST), Daejeon, 34141, Republic of Korea*

¶*these authors contributed equally to this work*

E-mail: nandk@kaist.ac.kr; lucio.isa@mat.ethz.ch

S1 SEM image of PDMS mold for capillary assembly

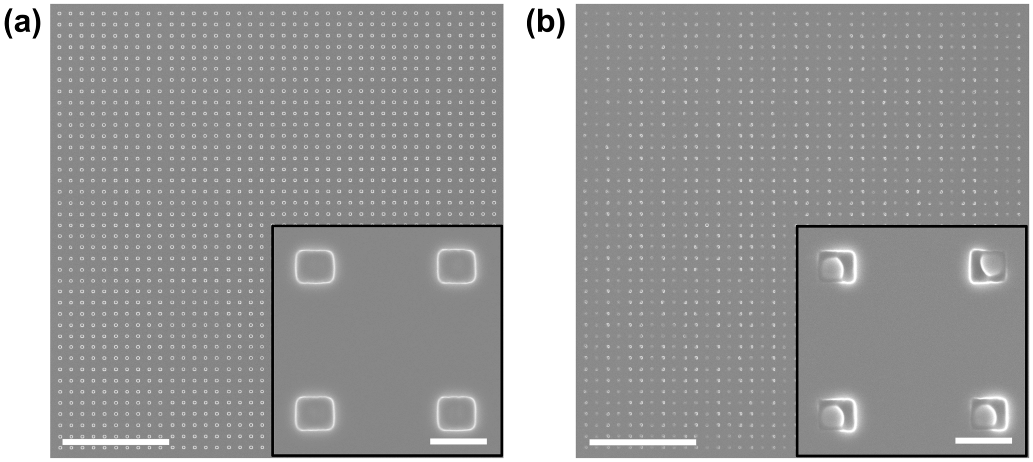

Figure S1: SEM images of 40×40 rectangular traps in PDMS (**a**) before CAPA (**b**) and after CAPA. Insets are enlarged images. Scale bars are 50 µm, and inset scale bars are 2 µm.

S2 Extra Statistical Tests

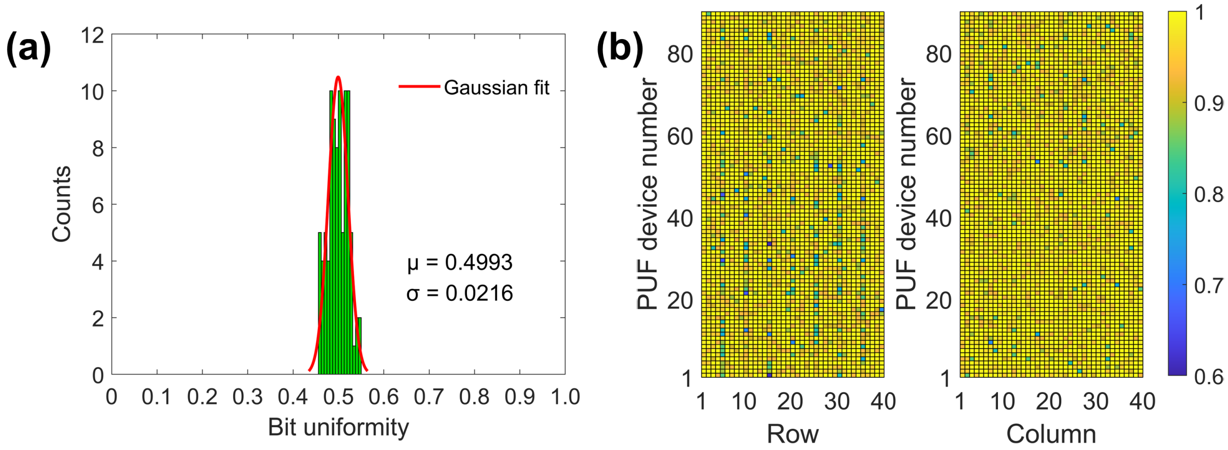

Figure S2: (**a**) Bit uniformity as a histogram with a Gaussian fit to visualize the distribution. (**b**) Alternative presentation of the entropy along row (x-axis) and column (y-axis) for each PUF. The average entropy values along rows and columns for overall 90 PUFs are 0.9789 ± 0.0298 and 0.9769 ± 0.0349, respectively.

S3 Detailed derivation of the probability of reproducing identical PUFs for a non-ideal distribution of colloid types

For the two-color case, i.e., green and black, we define the probability of having a green colloid, p, and then correspondingly the probability of having a black one as $(1-p)$. Our token is presented as a 2D system but for the measurement of its uniqueness, we can think of it as a single string of all rows stitched after each other, giving a total length $N = L \times L$.

For a single deposition the probability that a second sample is the same as the first one corresponds to the probability that it is green twice plus the one that it is black twice, so

$$p^2 + (1-p)^2 = p^2 + 1 - 2p - p^2 = 2p^2 - 2p + 1 \tag{1}$$

which gives us the expected outcome of 50% for the case of $p = 1/2$. The probability that a sequence of N depositions is the same is the factorization of the probability that each of these depositions is the same, so

$$P_2(N) = (2p^2 - 2p + 1)^N, \tag{2}$$

which for $p = 1/2$ gives us the trivial case $1/2^N$.

For three colloids we need to introduce the independent values p_1 for green, and p_2 for blue, which then gives us $(1-p_1-p_2)$ for black. Here again, the probability can be calculated as

$$P_3(1) = p_1^2 + p_2^2 + (1 - p_1 - p_2)^2 = 2(p_1^2 + p_2^2) + 2p_1p_2 - 2(p_1 + p_2) + 1, \tag{3}$$

which for $p_1 = 1/3 = p_2$ gives $P_3(1) = 1/3$. For an N-sequence this gives;

$$P_3(N) = (2(p_1^2 + p_2^2) + 2p_1p_2 - 2(p_1 + p_2) + 1)^N \tag{4}$$

The minimum is at $p_1 = 1/3 = p_2$, but in case of an unequal distribution of more than two colloids, there is an infinite amount of ways to distribute p_1 and p_2. In Figure S3, we plot the probability of filling a single trap ($N = 1$) twice with the same type of particle out of a three-particle-type suspension, i.e., corresponding to a 3-color PUF. It is clear that the lowest probability of reproducing the pixel value is around $p_1 = p_2$ for any value of p_3, and has a global minimum for $p_1 = p_2 = p_3 = 0.33$ (Figure S3a). Furthermore, by fixing the value of p_3, one can see that the probability to fill one single trap with the same color twice is quadratic in p_1 (or respectively $p_2 = 1 - p_3 - p_1$), and shows a minimum for $p_1 = p_2 = (1 - p_3)/2$ (Figure S3b). Correspondingly, the variation of such probability is the smallest around the minimum, implying that the assumption that $p_1 = p_2$ is robust against variation of their values. For example, we can consider the case of a fixed value for $p_3 = 0.5$, where in the case of $p_1 = p_2 = 0.25$ the probability of filling the trap twice with the same color is 0.375. A 5% deviation from this optimal ratio, e.g. $p_1 = 0.2$ and $p_2 = 0.3$, would increase this probability to 0.38, corresponding to a 1.3% increase.

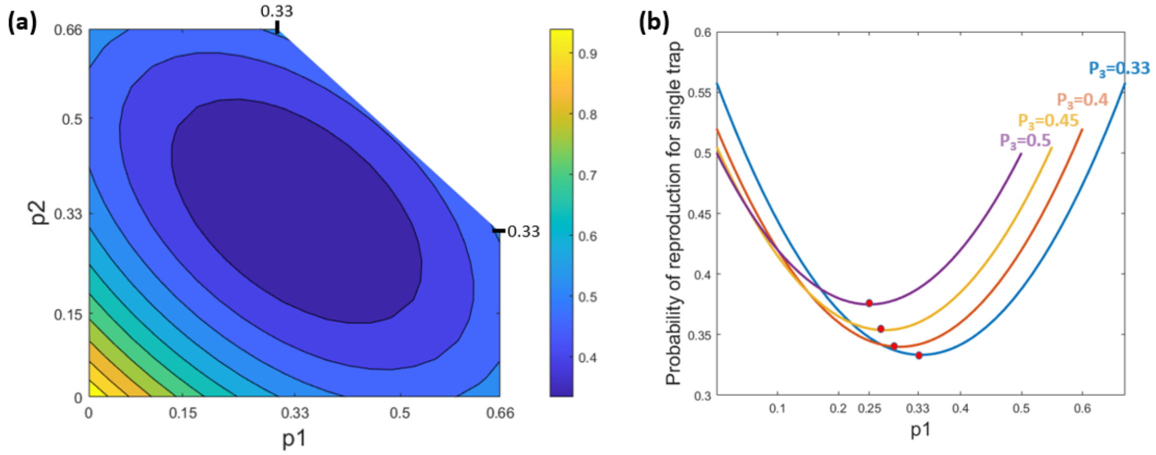

Figure S3: **(a)** 2D plot of the probability to reproduce the filling of a single trap (equation 3) for all values of p_1 and p_2, limited to $p_1 + p_2 = 1$. **(b)** Plot of the probability to reproduce the filling of a single trap for fixed values of p_3 which fixes $p_2 = 1 - p_1 - p_3$. The minimum values are presented with a red dot.

In our case, we have an equal distribution of the different types of colloids in solution. So, the most likely cause of inequality is when there are traps left empty leading to an

over-representation of the non-fluorescent areas. Therefore, we can approximate our case by having a single type of colloid, e.g., the black one, to be over-represented and equally distribute the other probabilities, i.e., mathematically setting $p_1 = p = p_2$, which simplifies our system to

$$P_3^{simp}(N) = (4p^2 + 2p^2 - 4p + 1)^N. \tag{5}$$

For four colloids we follow the same idea including p_3 for red, which gives

$$P_4(N) = (2(p_1^2 + p_2^2 + p_3^2) - 2(p_1 + p_2 + p_3) + 2(p_1p_2 + p_1p_3 + p_2p_3) + 1)^N, \tag{6}$$

and for our simplified case with $p_1 = p_2 = p_3 = p$;

$$P_4^{simp}(N) = (12p^2 - 6p + 1)^N \tag{7}$$

S4 Digitization process of fluorescence images

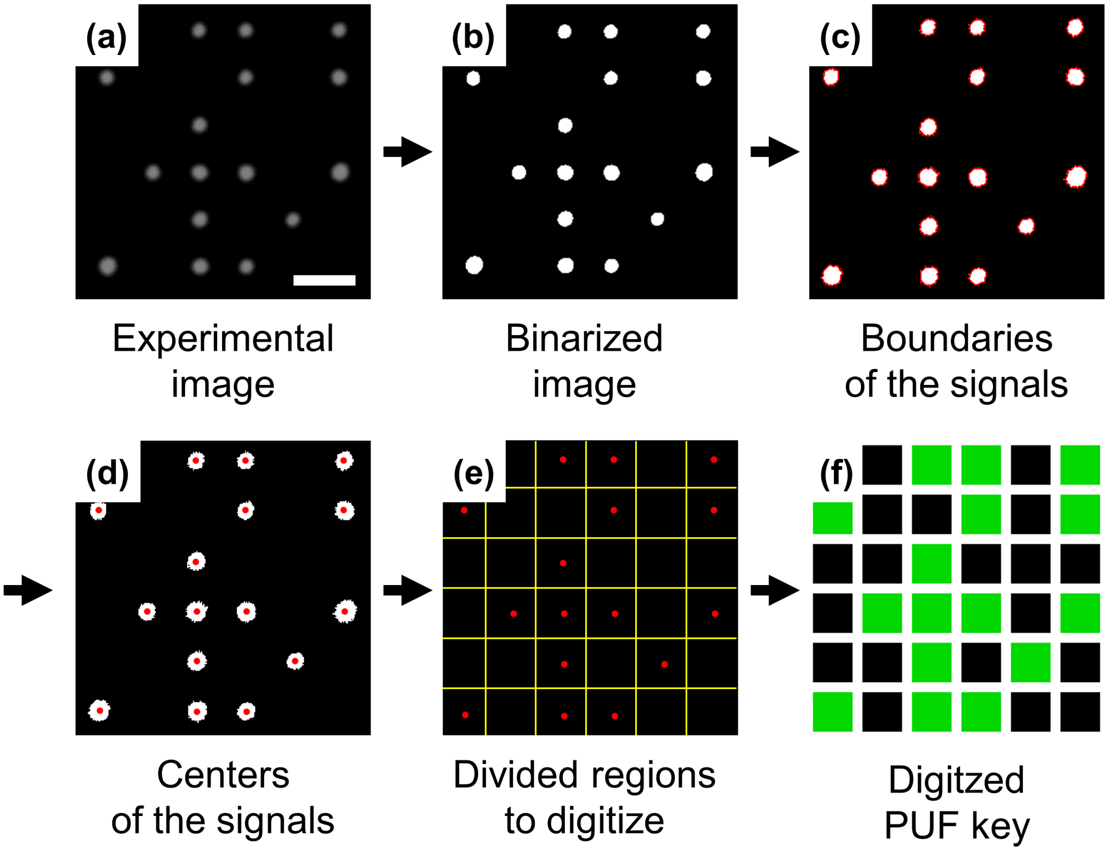

Figure S4: Digitization process of the fluorescence microscope images to generate PUF-key. (**a**) The Fluorescence microscopy image is loaded. (**b**) The image is binarized to see the fluorescence signal more clearly. (**c**) Boundaries of the fluorescent regions are defined. (**d**) The centers of the fluorescent regions are identified. (**e**) The image is divided into $L \times L$ regions to digitize the image. (**f**) A digitized PUF-key is obtained. Scale bar is 5 μm.

All digitization processes of the fluorescence images are conducted by custom-written Matlab codes. The fluorescence microscope image is loaded in the Matlab software and the contrast of the image is adjusted by "imadjust" function to extract the fluorescence signal more clearly. Noise in the image is eliminated by "bwareaopen", "bwconncomp", "region-props", "labelmatrix", and "ismember" functions. After this, the processed image is binarized. The boundary of the fluorescence signal is defined using "bwboundaries" function, which also gives as an output the location of the centers of the binarized regions. Finally, the PUF-key is obtained as a $L \times L$ digitized key by dividing the image into $L \times L$ regions and

binarizing it to 1 if there is a center of the fluorescence signal in each region and 0 if not. Each color of the fluorescence signal (red, green, and blue) are merged to obtain PUF-keys for multiple types of colloids. Note that only a 6×6 traps of the token is shown to present the digitization process clearly.

S5 Test of the authentication under artificial imaging noise

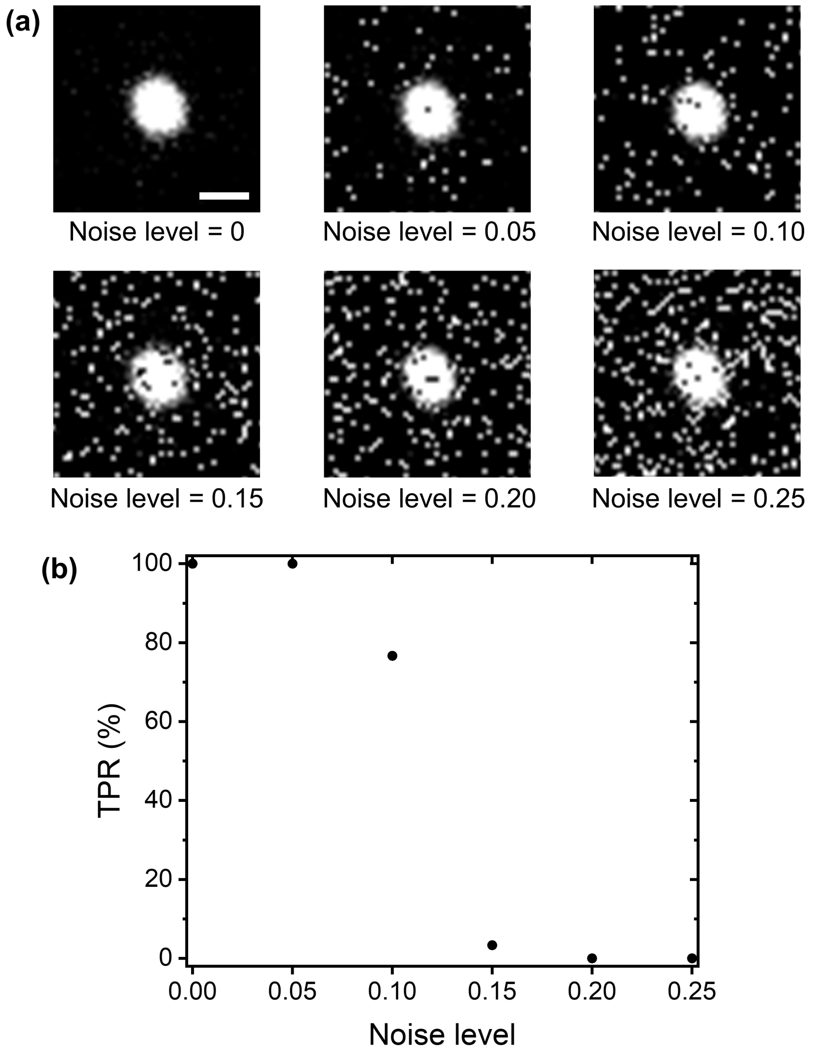

Figure S5: (**a**) Fluorescence microscope images of an individual particle deposited using CAPA for different levels of added digital noise. Noises levels are applied from 0 to 0.25 with steps of 0.05 using the "salt & pepper" model in "imnoise" function in Matlab. (**b**) True positive rate (TPR) for 90 PUF-tokens depending on the noise level to show the effect of artificial noise on the validation process. The threshold value used for authentication is 0.0278. Scale bar is 1 µm.

S6 Stability tests of PUFs encased in PDMS

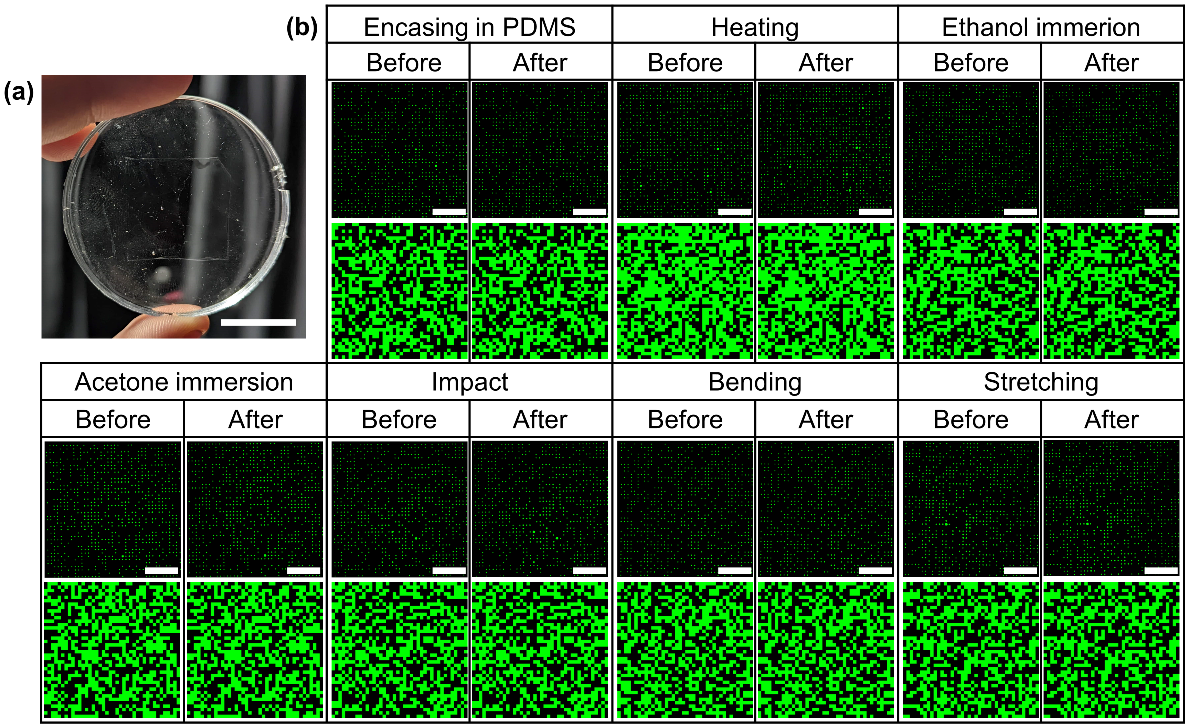

(c)

Test	Encasing in PDMS	Heating	Ethanol immersion	Acetone immersion	Impact & pressure	Bending	Stretching
Intra-HD	0	6.25×10^{-4}	6.25×10^{-4}	0	0	0	0
Result of authentication	True	True	True	True	True	True	True

Figure S6: (**a**) Photograph of the token encased in PDMS. (**b**) Fluorescence images and corresponding PUF-keys before and after various tests: encasing in PDMS, heating at 200 °C for 30 min, ethanol and acetone immersion for 30 min, impact & pressure of 4 N/µm^2, 100 bending cycles, and 20% stretching. (**c**) Intra-HD and results of authentication after each test. The threshold value used for authentication is 0.0278. Scale bar in (a) is 2 cm, (b) is 50 µm.

S7 Photostability test under UV exposure

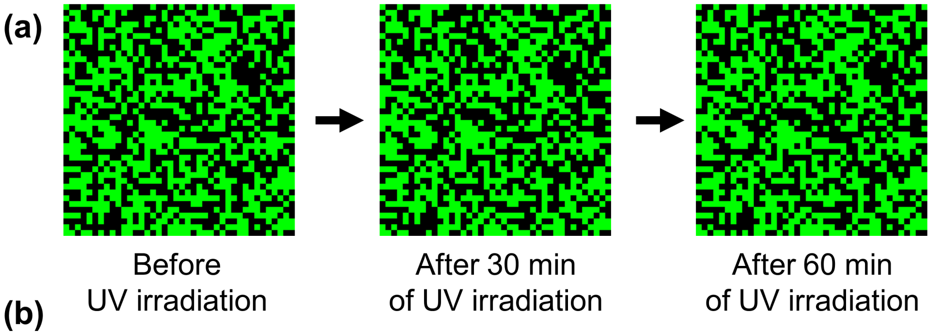

(b)

UV irradiation time	10 min	20 min	30 min	40 min	50 min	60 min
Intra-HD	0.0025	0.0138	0.0131	0.0063	0.0106	0.0100
Result of authentication	True	True	True	True	True	True

Figure S7: (**a**) PUF-key images after UV light irradiation of different durations. The power density and wavelength of the UV light used for the test are 61 mW/cm^2 and 365 nm, respectively. (**b**) Intra-HD and results of authentication as a function of UV light irradiation time. The threshold value used for authentication is 0.0278.

S8 Photostability test under daylight

(a)

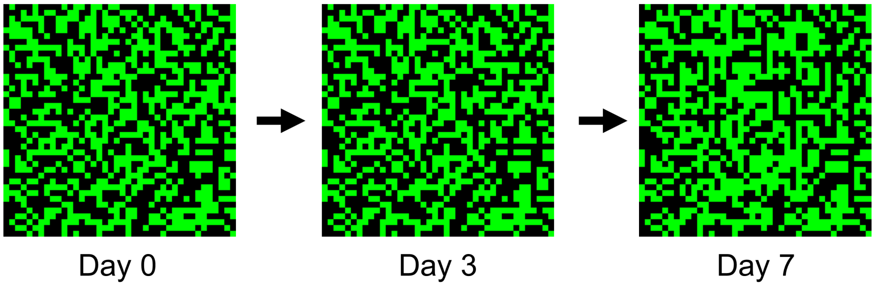

(b)

**Elapsed time**	**1 day**	**2 day**	**3 day**	**4 day**	**5 day**	**6 day**	**7 day**
Intra-HD	0	0.010	0.0075	0.035	0.0594	0.0388	0.0931
Result of authentication	True	True	True	False	False	False	False

Figure S8: (**a**) PUF key images after exposure to daylight for different time intervals, and (**b**) corresponding Intra-HD and results of authentication. The threshold value used for authentication is 0.0278.
